# Combining bioinformatics and machine learning to analyze and validate sepsis-related cell senescence genes and potential drugs

**DOI:** 10.1080/0886022X.2026.2667584

**Published:** 2026-05-31

**Authors:** Shuaijie Pei, Deqiang Li, Xiaoli Yu, Xiaofan Huang, Jianfeng Liu, Yu Song, Lin Zhu, Jiatian Cui, Yan Cui, Keliang Xie

**Affiliations:** ^a^ Department of Critical Care Medicine, Tianjin Medical University General Hospital, Tianjin, China; ^b^ Department of Anesthesiology, Tianjin Medical University General Hospital, Tianjin, China; ^c^ Department of Anesthesiology, Tianjin Medical University Baodi Hospital, Tianjin, China; ^d^ Department of Pathogen Biology, School of Basic Medical Sciences, Tianjin Medical University, Tianjin, China

**Keywords:** Sepsis, senescence, bioinformatics analysis, machine learning, fenofibrate

## Abstract

Sepsis is a life-threatening organ malfunction induced by the host’s abnormal reaction to infection. Sepsis can induce cellular senescence, thereby exacerbating tissue damage and organ dysfunction. However, the key biomarkers of cellular senescence and the corresponding targeted therapeutics in sepsis remain unknown. This study identified eight differentially expressed senescence-related genes, including TXN, CDKN1C, UTP6, BCL11B, SMAD3, ITPKB, PRPF19, and BCL2, through bioinformatics analysis and machine learning. These hub genes had good diagnostic performance for sepsis. Six hub genes showed consistent trends in the validation set and experimental samples with those in the training set. Immunoinfiltration analysis showed that eosinophils, macrophages M1, macrophages M2, NK cells activated, NK cells resting, T cells CD8, and Tregs were substantially linked with all hub genes. A large number of targeted compounds or drugs were obtained from the DSigDB database based on hub genes. These drugs primarily interacted with CDKN1C, BCL2, and SMAD3. The binding energies of fenofibrate with these target proteins were less than −5.0 kcal/mol. In both *in vivo* and *in vitro* models of sepsis-induced acute kidney injury (AKI), fenofibrate has been observed to alleviate senescence and inflammation. In conclusion, the present study identified eight DE-SRGs associated with sepsis and validated their diagnostic efficacy. And, fenofibrate might exert anti-inflammatory and anti-senescence effects in sepsis-induced AKI by regulating senescence genes, shedding fresh light on sepsis treatment.

## 1. Introduction

Sepsis is a life-threatening organ malfunction induced by the host’s abnormal reaction to infection according to sepsis-3 [1]. Sepsis has been declared a global health problem by the World Health Organization (WHO). According to a research based on the Global Burden of Disease (GBD) database, there were around 48.9 million sepsis cases globally in 2017, of which 11 million resulted in death, accounting for 19.7% of all fatalities worldwide [2]. Only around half of patients with sepsis will fully recover, and 17% of those who survive will develop post-sepsis syndrome (PSS), which significantly raises the risk of reinfection and mortality [3,4]. The etiology of sepsis is complex and the condition changes rapidly, leaving early identification and therapy challenging. Acute kidney injury (AKI) occurs early in sepsis and is characterized by impaired filtration and clearance functions. Multinational AKI-Epidemiologic Prospective Investigation (EPI) study reveals that the incidence of AKI among patients on their first day in the ICU is 57.3%, with 21% of these cases occurring in sepsis patients [5]. AKI is associated with the development of chronic kidney disease (CKD), longer hospital stays, greater resource consumption, higher early and late mortality, and other long-term systemic complications [6,7]. As a result, it is critical to continue investigating the underlying causes of AKI and developing innovative treatments.

Cell senescence is an irreversible state of cell cycle arrest in which proliferating cells become resistant to pro-growth stimuli [8]. Hayflick and Moorhead initially described this phenomenon in the early 1960s [9]. Senescent cells are characterized by morphological and metabolic changes, chromatin remodeling, altered gene expression, and the emergence of a pro-inflammatory phenotype called senescence associated secretory phenotype (SASP) [10,11]. Cellular senescence is not only a hallmark of aging, but it also plays a critical role in organism development, tissue remodeling and repair, tissue homeostasis maintenance, and wound healing [12,13]. Sepsis can trigger large-scale inflammatory responses, oxidative stress, and mitochondrial dysfunction, all of which may induce cellular senescence, leading to tissue damage and organ dysfunction [14,15]. It is of great clinical significance to elucidate the interaction between cell senescence and sepsis.

High-throughput sequencing may extract practically all transcripts from tissues, allowing for thorough gene function study and the discovery of disease molecular mechanisms. Machine learning is a critical subject in bioinformatics that can be used for data mining, analysis, and model development. Machine learning algorithms can identify trends in high-throughput data and help search for genes with biological value. This study aims to use high-throughput sequencing data, combined with bioinformatics analysis and machine learning, to thoroughly elucidate the genetic markers and potential therapeutic drugs for patients with sepsis, thereby providing a basis for future research.

## 2. Materials and methods

### 2.1. Acquisition of data

The high-throughput sequencing data was obtained from the Gene Expression Omnibus (GEO) database (https://www.ncbi.nlm.nih.gov/geo/). The search keywords were (‘sepsis’[MeSH Terms] OR ‘sepsis’[All Fields]) AND ‘Homo sapiens’[porgn] AND (‘gse’[Filter] AND ‘Expression’ profiling by ‘array’[Filter]). Dataset GSE65682 (platform: GPL13667, Affymetrix Human Genome U219 Array, Santa Clara, CA) and GSE28750 (platform: GPL570, Affymetrix Human Genome U133 Plus 2.0 Array, Santa Clara, CA) were used for subsequent analyses. The dataset GSE65682 included 760 sepsis patients and 42 healthy controls. Dataset GSE28750 included 10 sepsis patients and 20 healthy controls. Blood samples from patients with sepsis were collected within 24 h after ICU admission in both datasets. The dataset GSE65682 was used as the training set and the other dataset was used as the validation set. The cell senescence gene set was obtained from the CellAge database (https://genomics.senescence.info/cells/).

### 2.2. Identification of differentially expressed genes

First, the gene expression matrix was generated according to the probe expression matrix and the platform data of the probe. The data were log2 normalized to generate a normalized expression matrix. Differentially expressed genes (DEGs) between sepsis group and healthy control group were identified by ‘limma’ package. The filter conditions for DEGs were |log2FC| > 0.585 (corresponding fold change was 1.5) and adjusted *p* value < 0.05. Heatmaps were created with the ‘pheatmap’ package, while volcano plots were created with the ‘ggplot2’ package.

### 2.3. Weighted gene co-expression network analysis

Weighted gene co-expression network analysis (WGCNA) was a bioinformatics method used to describe gene patterns between different samples. It clustered genes with similar expression patterns and analyzed the relationship between modules and specific traits. Potential abnormal samples could be identified and excluded by constructing the clustering dendrogram. By using the scale-free topology criterion, a soft-thresholding power (*β*) was chosen when the scale-free topology index (*R*^2^) was more than 0.85. The adjacency matrix was calculated and transformed into a topological overlap matrix (TOM), reflecting the network connectivity between genes. The TOM-based dissimilarity metric was used to cluster genes in a hierarchical fashion. A dynamic tree cut was used to identify gene modules, with a minimum module size of 50 genes.

Each gene module’s link with sepsis was determined using the Pearson correlation test. The module with a significant correlation (*p* < 0.05) was deemed clinically meaningful.

Gene significance (GS), which was the absolute value of the correlation between each gene’s expression and the trait of interest, was computed in order to quantify the association between individual genes and clinical traits. Module membership (MM) was defined as the correlation between each gene’s expression and the module eigengene, representing the degree to which a gene belongs to a particular module.

MM and GS were used to evaluate the significance of the module.

### 2.4. Enrichment analysis

Enrichment analysis was a technique that linked genes with related functions to signaling pathways or biological traits. Using the Gene Ontology (GO) database (http://geneontology.org/), functional enrichment analysis of the chosen genes was carried out in this study. Genes were described consistently according to their molecular function (MF), biological process (BP), and cellular components (CCs). The pathway enrichment analysis was carried out through Kyoto Encyclopedia of Genes and Genomes (KEGG) database. Gene set enrichment analysis (GSEA) was used to assess the distribution trend of genes in *a priori* defined set of genes in a phenotypic correlation sequence to determine their contribution to phenotype. GSEA took into account genes that were less different in the whole genome. The ‘clusterProfiler’ package was used for enrichment analysis, while ‘org.Hs.eg.db’ package was utilized for gene ID conversion. The result was visualized by ‘enrichplot’ and ‘ggplot2’ packages.

### 2.5. Protein–protein interaction network analysis

Protein–protein interaction (PPI) network analysis could be used to describe the interactions between proteins. Interacting Genes were described via the Search Tool for the Retrieval of Interacting Genes (STRING) online database (http://string-db.org). A protein was represented by each node. The interaction between two proteins was indicated by the lines connecting the nodes. Protein interactions required a minimum interaction score of more than 0.4. Sources of evidence for interaction included Textmining, Experiments, Databases, Co expression, Neighborhood, Gene Fusion, and Co-occurrence. The free nodes indicated that no protein with which the protein interacted was retrieved from the database, and the free nodes were hidden. The PPI network was further visualized via Cytoscape software, with red circles representing up-regulated proteins and green circles representing down-regulated proteins.

### 2.6. Screening and validation of hub genes

First, the DEGs filtered by ‘limma’ package, the core module genes screened by WGCNA and the cell senescence gene set were intersected. Intersection genes were further selected employing three machine learning algorithms: least absolute shrinkage and selection operator (LASSO) regression, random forests (RFs), and support vector machine-recursive feature elimination (SVM-RFE). LASSO regression was implemented through ‘glmnet’ package, which built a linear model with penalty terms to shrink coefficients of less relevant features to zero. The regularization parameter (*λ*) was optimized by 10-fold cross-validation, minimizing the mean squared error in the training dataset. Features with non-zero coefficients were selected as potential biomarkers. The RF algorithm was performed by the ‘randomForest’ package. SVM-RFE was a wrapper-based feature selection method for iteratively removing less important features while maximizing the performance of support vector machine (SVM) classifiers. The optimal feature subset was determined by cross-validation to maximize the classification accuracy. Hub genes were the intersection genes obtained by three machine learning methods. A logistic regression model was constructed on the basis of hub genes. The predictive performance of individual genes and logistic regression models was evaluated by receiver operating characteristic (ROC) curve and area under the curve (AUC) in the training set and validation set, respectively.

### 2.7. Analysis of immune infiltration

The relative expression of 22 types of immune cells in the sample was obtained with the ‘CIBERSORT’ package. The packages ‘ggplot2’, ‘corrplot’, and ‘pheatmap’ were used to plot the correlation diagram between immune cells and core genes as well as immune cells.

### 2.8. Screening of potential therapeutic compounds and molecular docking

Potential targeted compounds were obtained in the DSigDB database (https://dsigdb.tanlab.org/DSigDBv1.0/). The 3D structures of the compounds were obtained from PubChem (https://pubchem.ncbi.nlm.nih.gov/). The ligand structure was then converted to PDBqt format using AutoDockTools and hydrogen and charge were added. The protein structures encoded by hub genes were retrieved from the RCSB protein database (PDB, http://www.pdb.org/). The protein structures predicted by AlphaFold were used for proteins lacking experimentally resolved structures. The protein structures were cleaned by removing water molecules, adding polar hydrogens, and assigning charges using AutoDockTools. AutoDock Vina was used for docking simulation to predict the binding conformation and affinity of ligands and target proteins. The binding energy of each protein–ligand complex was calculated in kcal/mol, and complexes less than −5.0 kcal/mol were considered stable. The docking pose with the lowest binding energy was selected for further analysis, and PyMOL (v3.1.1) and Ligplot (v2.2.5) were used to visualize intermolecular interactions, including hydrogen bonding, hydrophobic contact, and other interactions. Key residues involved in binding were identified to infer the molecular basis of the interaction.

### 2.9. Animal experiments

This study used male C57BL/6J mice aged 6–8 weeks and weighing 20–25 g, provided by SPF Biotechnology Co., Ltd. (Beijing, China). The animal license number was SCXK(JING)2019-0010. Mice were kept in a specific pathogen free (SPF) environment with the humidity of around 40% and a temperature range of 20–22 °C. Mice were given unrestricted access to food and water, as well as 12 h of light and 12 h of darkness every day. The mice were divided into four groups: Sham group, Sham + fenofibrate (F) group, cecal ligation and puncture (CLP) group, and CLP + F group. Before the experiment began, the mice were randomly assigned to each group using a random number table.

Mice in the CLP group underwent CLP to establish an *in vivo* sepsis model. Two percent isoflurane (R510-22-10, RWD, Shenzhen, China) was utilized to keep anesthesia throughout surgery. The fur on the mice’s belly was stripped off using hair removal cream, after which the skin was cleaned with saline. Make a 1.0 cm incision along the abdomen’s midline to expose the cecum. Ligate 50% of the cecum and pierce it with a 21 G needle, releasing a tiny quantity of feces. Return the cecum to the abdominal cavity and stitch the wound. Mice were given butorphanol (0.05 mg/kg) intraperitoneally to alleviate pain following surgery. To resuscitate the mice and replenish surgical fluid losses, prewarmed sterile saline (37 °C) was administered subcutaneously at a dose of 5 mL per 100 g body weight (approximately 1.0–1.25 mL per mouse), followed by placement of the mice on a 37 °C warming blanket. Mice in Sham group merely received the open abdomen and cecum release surgery. Mice in Sham + F group and CLP + F group received fenofibrate (100 mg/kg/d; GC12250, GlpBio, Montclair, CA) orally for two weeks before undergoing Sham surgery or CLP. Twenty-four hours post-surgery, mice were euthanized. Anesthesia was induced by inhalation of a 4% isoflurane–oxygen mixture. Once mice reached deep anesthesia (approximately 1–2 min later), a lethal dose of sodium pentobarbital (150 mg/kg; P3761, Sigma-Aldrich, St. Louis, MO) was rapidly administered via intraperitoneal injection. Subsequent experiments were conducted after confirming animal death. All procedures were performed by trained and qualified research personnel. The Tianjin Medical University Experimental Animal Management Committee accepted all experimental protocols (IRB2025-DW-99).

### 2.10. Cell experiments

Human proximal tubule epithelial cells (HK-2; CL-0109, Procell, Wuhan, China) were obtained from Wuhan Procell Co., Ltd. (Wuhan, China) and cultured in HK-2 Cell Complete Medium (CM-0109, Procell, Wuhan, China) at 37 °C in a humidified atmosphere containing 95% air and 5% CO_2_. The following experiment involved dividing HK-2 cells into four groups: control group, control + F group, LPS group, and LPS + F group.

The control + F group received 100 μM fenofibrate for 24 h, while the LPS group received 1 μg/mL LPS (L8880, Solarbio, Beijing, China) for the same duration. The LPS + F group received both LPS and fenofibrate for 24 h. The LPS + F group was simultaneously treated with the above-mentioned concentration of LPS and fenofibrate for 24 h. The 24-h treatment duration was selected based on established models where it represents the peak inflammatory response to LPS and aligns with the timeframe demonstrated for fenofibrate’s cytoprotective efficacy in HK-2 cells [16,17].

### 2.11. Examination of renal function and histology

Approximately, 0.5 mL of blood was collected from each mouse 24 h after CLP.

After one hour at room temperature, blood samples were centrifuged at 3,000 × *g* for 15 min to obtain serum. The colorimetric method was used to measure the concentrations of serum creatinine (E-BC-K188-M, Elabscience, Wuhan, China) and urea nitrogen (E-BC-K183-M, Elabscience, Wuhan, China). The experiment was conducted strictly in accordance with the instructions. For histological examination, mice were first perfused with pre-cooled phosphate-buffered saline (PBS), and then kidney tissue was fixed in 4% paraformaldehyde. The fixed tissue was dehydrated with ethanol and embedded in paraffin, then cut into 5 μm sections for HE staining. The stained sections were observed using a NanoZoomer S360 microscope (Hamamatsu, Japan). The severity of tubular injury in kidney sections was assessed in a blinded manner. Histopathological changes were evaluated by calculating the percentage of damaged tubules per field, based on criteria including tubular necrosis, dilation, structural disruption, and cast formation. Tissue injury was scored on a scale of 0–4, where scores of 0, 1, 2, 3, and 4 correspond to 0%, <25%, 26–50%, 51–75%, and ≥76% of tubules damaged, respectively [18].

### 2.12. Enzyme-linked immunosorbent assay (ELISA)

After extracting mouse serum using the previously mentioned method, the levels of interleukin-1β (IL-1β; SEKM-0002, Solarbio, Beijing, China), interleukin-6 (IL-6; SEKM-0145, Solarbio, Beijing, China), and tumor necrosis factor-α (TNF-α; SEKM-0034, Solarbio, Beijing, China) in the serum were measured in accordance with the ELISA kit’s instructions.

### 2.13. SA-β-gal staining

Mice were sacrificed 24 h after CLP and perfused with pre-cooled PBS. The kidneys were removed, fixed in 4% paraformaldehyde, dehydrated with sucrose, embedded in OCT, and stored in a freezer at −80 °C. The kidneys were cut into 10 µm sections using the HM525NX cryostat (Thermo Fisher Scientific, Waltham, MA), after which the SA-β-Gal activity was detected according to the instructions provided with the SA-β-Gal kit (G1580, Solarbio, Beijing, China). For *in vitro* experiments, the activity of SA-β-Gal in the treated HK-2 cells was also detected in accordance with the instructions. Images were gathered using a NanoZoomer S360 microscope (Hamamatsu, Japan).

### 2.14. Western blotting

Grind kidney tissue thoroughly after adding RIPA lysis buffer (R0010, Solarbio, Beijing, China) and PMSF (P0100, Solarbio, Beijing, China) at a 100:1 ratio. Centrifuge the tissue lysate at 14,000 × *g* for 10 min at 4 °C, then transfer the supernatant to a new Eppendorf tube. The concentration of protein was determined using a BCA protein assay kit (PC0020, Solarbio, Beijing, China). Finally, add loading buffer to the supernatant and boil the protein sample at 100 °C for 10 min. The protein samples were subjected to 4–12% sodium dodecyl sulfate-polyacrylamide gel electrophoresis (SDS-PAGE), followed by transfer to a polyvinylidene difluoride (PVDF) membrane (FP39, Beyotime, Shanghai, China). The PVDF membranes were blocked with 5% skimmed milk at room temperature for two hours, subsequently undergoing an overnight incubation at 4 °C with the following antibodies: p53 (1:1,000; ab26, Abcam, Cambridge, UK), p16 (1:2,000; ab211542, Abcam, Cambridge, UK), and GAPDH (1:1,000; ab8245, Abcam, Cambridge, UK). After being incubated with the appropriate horseradish peroxidase-conjugated secondary antibodies – goat anti-rabbit IgG (1:5,000, S0001, Affinity, Beijing, China) and goat anti-mouse IgG (1:5,000, S0002, Affinity, Beijing, China) – the bands were exposed and imaged the following day using an AlphaView imaging system (ProteinSample, San Jose, CA) and enhanced chemiluminescence reagents (E-IRR308, Elabscience, Wuhan, China). Protein bands were examined for grayscale values using Image-J software (Bethesda, MD). Protein expression was shown by the target protein’s gray value divided by GAPDH.

### 2.15. Real-time quantitative PCR

The Animal Total RNA Isolation Kit (RE-03011, FOREGENE, Chengdu, China) was used to extract the total RNA from mice kidney tissues, and a reverse transcription kit (RT-01031, FOREGENE, Chengdu, China) was used to generate cDNA. Real-time quantitative polymerase chain reaction (RT-qPCR) was performed using the SYBR Green Premix Pro Taq HS qPCR Kit (AG, AG11701, Hunan, China). The study used 2^−ΔΔCt^ to measure relative quantities of hub genes, with GAPDH acting as the internal reference. The primer sequences, which Sangon Biotech (Shanghai, China) synthesized after finding them on the National Center for Biotechnology Information website (https://www.ncbi.nlm.nih.gov/), were displayed in Table 1.

**Table 1.** Primer sequences (mouse).

Gene	Primer sequences
SMAD3 (F)	GCTTTGAGGCTGTCTACCAGCT
SMAD3 (R)	GTGAGGACCTTGACAAGCCACT
BCL2 (F)	CCTGTGGATGACTGAGTACCTG
BCL2 (R)	AGCCAGGAGAAATCAAACAGAGG
TXN (F)	CAAATGCATGCCGACCTTCCAG
TXN (R)	GCTGGTTACACTTTTCAGAGCATG
ITPKB (F)	ACGCTACAACCAGATGGACGAC
ITPKB (R)	ATGTCCTTCCGCAAGCTAGGCT
CDKN1C (F)	AGCTGAAGGACCAGCCTCTCTC
CDKN1C (R)	ACGTCGTTCGACGCCTTGTTCT
PRPF19 (F)	GATTCTCGCTCTGGACCTGTGT
PRPF19 (R)	ACCACACTGGTGACCTTCTTGG
UTP6 (F)	AAGGCGAGTTGGCACGGATCAT
UTP6 (R)	GTCATCGGTGTGCAGAGCTTGA
BCL11B (F)	CTACTGTCACCCACGAAAGGCA
BCL11B (R)	GGCACGCAGAGGTGAAGTAATC
GAPDH (F)	AGGTCGGTGT-GAACG GATTTG
GAPDH (R)	GGGGTCGTTGATGGCAACA

F: forward primer (5′–3′); R: reverse primer (5′–3′).

### 2.16. Statistical analysis

All statistical analyses were conducted using GraphPad Prism 9.0 (GraphPad Software, San Diego, CA). Data were expressed as mean ± standard deviation (SD). The disparities between the two groups were examined using an independent samples *t*-test. Data from more than two groups were analyzed with one-way ANOVA followed by *post hoc* analysis with Tukey’s test. *p* < 0.05 indicated a significant difference.

## 3. Results

### 3.1. Identification of DEGs and hub gene modules in sepsis

**Figure 1.**
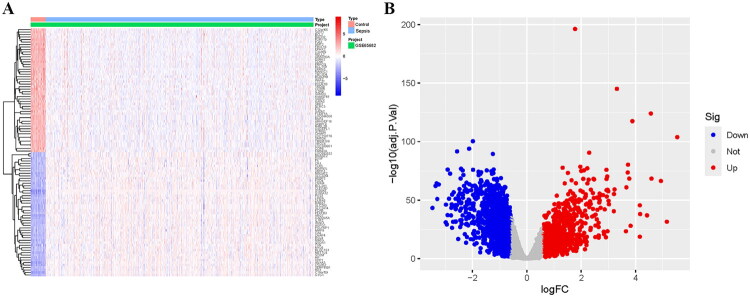
DEGs identification from a sepsis dataset. (A) Heatmap depicting gene expression profiles distinguishing sepsis patients from healthy controls. Each row represents a gene, and each column represents a sample. (B) Volcano plot of the 3,138 identified DEGs. Genes with significant up-regulation (log_2_FC > 0.585, adjusted *p* < 0.05) and down-regulation (log_2_FC < −0.585, adjusted *p* < 0.05) are highlighted in red and blue, respectively. Gray dots represent non-significant genes. DEGs: differentially expressed genes; FC: fold change.

**Figure 2.**
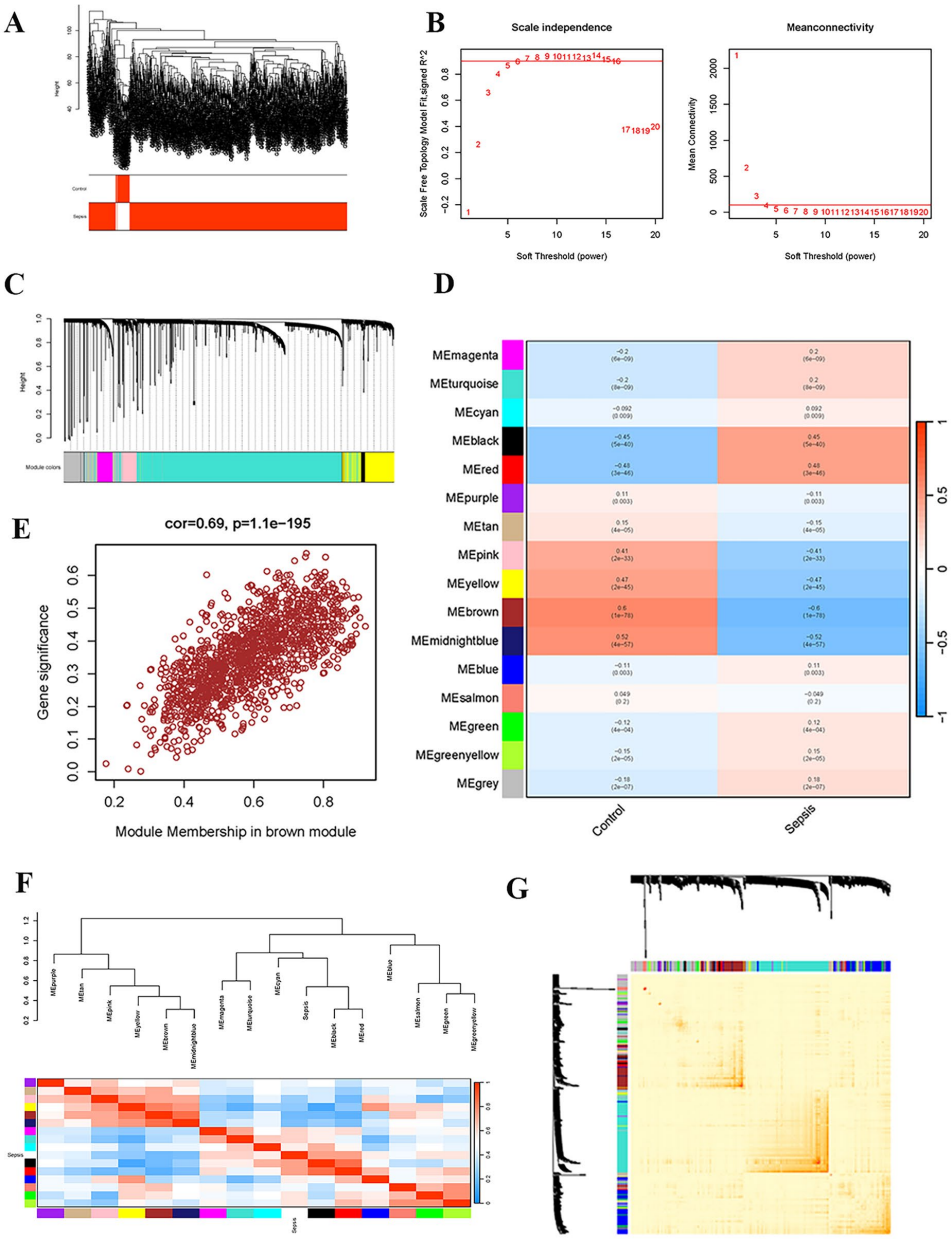
Weighted gene co-expression network analysis of the sepsis dataset. (A) Clustering dendrogram of 795 samples. (B) Determination of the optimal soft-thresholding power (*β* = 7). (C) Hierarchical clustering dendrogram of genes, with color bands below indicating assigned modules. (D) Module-trait relationships. The correlation coefficient and significance (*p* value) between each module eigengene and the sepsis phenotype are shown. (E) Scatter plot of GS for sepsis versus MM in the brown module. (F) Cluster dendrogram and the relationship between modules. (G) Gene co-expression network heatmap plot. GS: gene significance; MM: module membership.

The dataset GSE65682 obtained from the GEO database, which contained 760 sepsis patients and 42 controls, was used as the training set. A total of 3,138 DEGs could be obtained from the training set (Table S1), among which 1,384 were up-regulated and 1,754 were down-regulated (Figure 1). Then, weighted correlation network analysis (WGCNA) was used to determine the hub gene modules related to sepsis. The sample clustering dendrogram and the corresponding grouping are shown in Figure 2(A). The appropriate soft thresholding power *β* for establishing a scale-free network was determined by the scale-free fit index and mean connectivity, which was 7 (Figure 2(B)). The genes were eventually grouped into 16 modules using the hierarchical clustering analysis of the gene dendrograms and the dynamic branch cutting approach (Figure 2(C)). Figure 2(D) illustrates the link between the gene module and sample grouping. It was notable that the brown module (1,380 genes, *r* = −0.6, *p* = 1e − 78) showed a strong negative correlation with sepsis (Table S2). Moreover, there was also significance between the GS and MM of the brown module (cor = 0.69, *p* = 1.1e − 195) (Figure 2(E)). Figure 2(F) depicts the relationships between the modules. The brown module correlated strongly with the yellow and midnightblue modules, but very weakly with the red module. The association between genes is depicted in Figure 2(G).

### 3.2. Identification and enrichment analysis of differentially expressed senescence-related genes in sepsis

**Figure 3.**
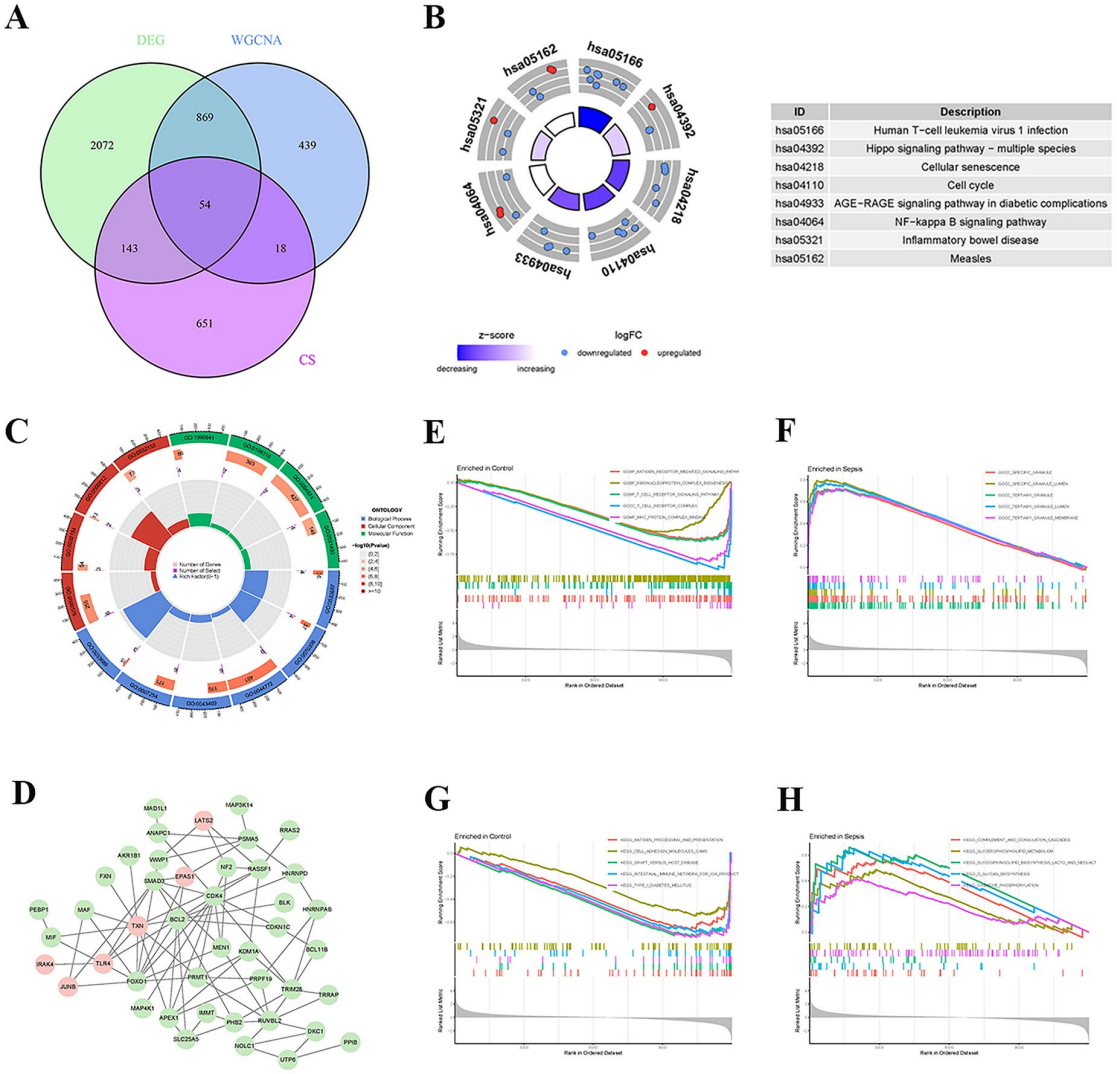
Functional enrichment analysis of DE-SRGs in sepsis. (A) The Venn diagram showing the intersection genes obtained from DEGs, the genes screened by WGCNA and the cell senescence gene set. (B) Circos plot of the top enriched KEGG pathways for the 54 DE-SRGs. (C) Circos plot showing the top enriched GO terms for the DE-SRGs in BP, CC, and MF categories. (D) PPI network of the DE-SRGs constructed using the STRING database. Node colors indicate expression changes: red for up-regulated and green for down-regulated genes. (E, F) GSEA plots of the top five GO terms significantly enriched in the (E) control group and (F) sepsis group, respectively. (G, H) GSEA plots of the top five KEGG pathways significantly enriched in the (G) control group and (H) sepsis group, respectively. DE-SRGs: differentially expressed senescence-related genes; DEGs: differentially expressed genes; WGCNA: weighted gene co-expression network analysis; KEGG: Kyoto Encyclopedia of Genes and Genomes; GO: Gene Ontology; BP: biological process; CC: cellular component; MF: molecular function; PPI: protein–protein interaction; GSEA: gene set enrichment analysis.

To identify differentially expressed senescence-related genes (DE-SRGs) in sepsis, 866 SRGs were retrieved from the CellAge database (https://genomics.senescence.info/cells/). Fifty-four DE-SRGs were determined using Venn diagrams based on difference analysis and WGCNA (Figure 3(A), Table S3). The KEGG circos plot demonstrated that DE-SRGs were considerably enriched in pathways including ‘Human T-cell leukemia virus 1 infection’, ‘Hippo signaling pathway-multiple species’, ‘Cellular senescence’, ‘Cell cycle’, ‘AGE–RAGE signaling pathway in diabetic complications’, ‘NF-kappa B signaling pathway’, ‘Inflammatory bowel disease’ as well as ‘Measles’ (Figure 3(B), Table S4). GO analysis indicated significant enrichment of DE-SRGs in the following BPs: ‘osteoblast proliferation’, ‘lens fiber cell differentiation’, ‘mitotic cell cycle phase transition’, ‘negative regulation of MAPK cascade’, ‘JNK cascade’, and ‘negative regulation of osteoblast proliferation’. The GO analysis also revealed significant enrichment in CCs, including ‘RNA polymerase II transcription regulator complex’, ‘cleavage furrow’, ‘Swr1 complex’, and ‘cell division site’, as well as in MFs, such as ‘promoter-specific chromatin binding’, ‘protein serine kinase activity’, ‘protein serine/threonine kinase activity’, and ‘chromatin DNA binding’ (Figure 3(C), Table S5). The PPI network demonstrated the potential interaction between DE-SRGs (Figure 3(D), Table S6). The red circles indicated up-regulated genes, whereas the green circles represented down-regulated genes. BCL2, FOXO1, and TXN all interacted with numerous genes.

This study also used GSEA to illustrate gene functions more completely. GO_GSEA revealed that the genes in the control group were mostly engaged in ‘GOBP_ANTIGEN_RECEPTOR_MEDIATED_SIGNALING_PATHWAY’, ‘GOBP_RIBONUCLEOPROTEIN_COMPLEX_BIOGENESIS’, ‘GOBP_T_CELL_RECEPTOR_SIGNALING_PATHWAY’, ‘GOCC_T_CELL_RECEPTOR_COMPLEX’, and ‘GOMF_MHC_PROTEIN_COMPLEX_BINDING’, while genes in the sepsis group were mainly involved in ‘GOCC_SPECFIC_GRANULE’, ‘GOCC_SPECIFIC_GRANULE_LUMEN’, ‘GOCC_TERTIARY_GRANULE’, ‘GOCC_TERTIARY_GRANULE_LUMEN’, and ‘GOCC_TERTIARY_GRANULE_MEMBRANE’ (Figure 3(E,F)). According to KEGG_GSEA, genes in the control group were largely enriched in pathways such as ‘KEGG_ANTIGEN_PROCESSING_AND_PRESENTATION’, ‘KEGG_CELL_ADHESION_MOLECULES_CAMS’, ‘KEGG_GRAFT_VERSUS_HOST_DISEASE’, ‘KEGG_INTESTINAL_IMMUNE_NETWORK_FOR_IGA_PRODUCT’, and ‘KEGG_TYPE_I_DIABETES_MELLITUS’, whereas genes in the sepsis group were mainly enriched in pathways such as ‘KEGG COMPLEMENT AND COAGULATION CASCADES’, ‘KEGG_GLYCEROPHOSPHOLIPID_METABOLISM’, ‘KEGG_GLYCOSPHINGOLIPID_BIOSYNTHESIS_LACTO_AND_NEOLACT’, ‘KEGG_O_GLYCAN BIOSYNTHESIS’, and ‘KEGG OXIDATIVE PHOSPHORYLATION’ (Figure 3(G,H)).

### 3.3. Screening for hub DE-SRGs with diagnostic value using machine learning

**Figure 4.**
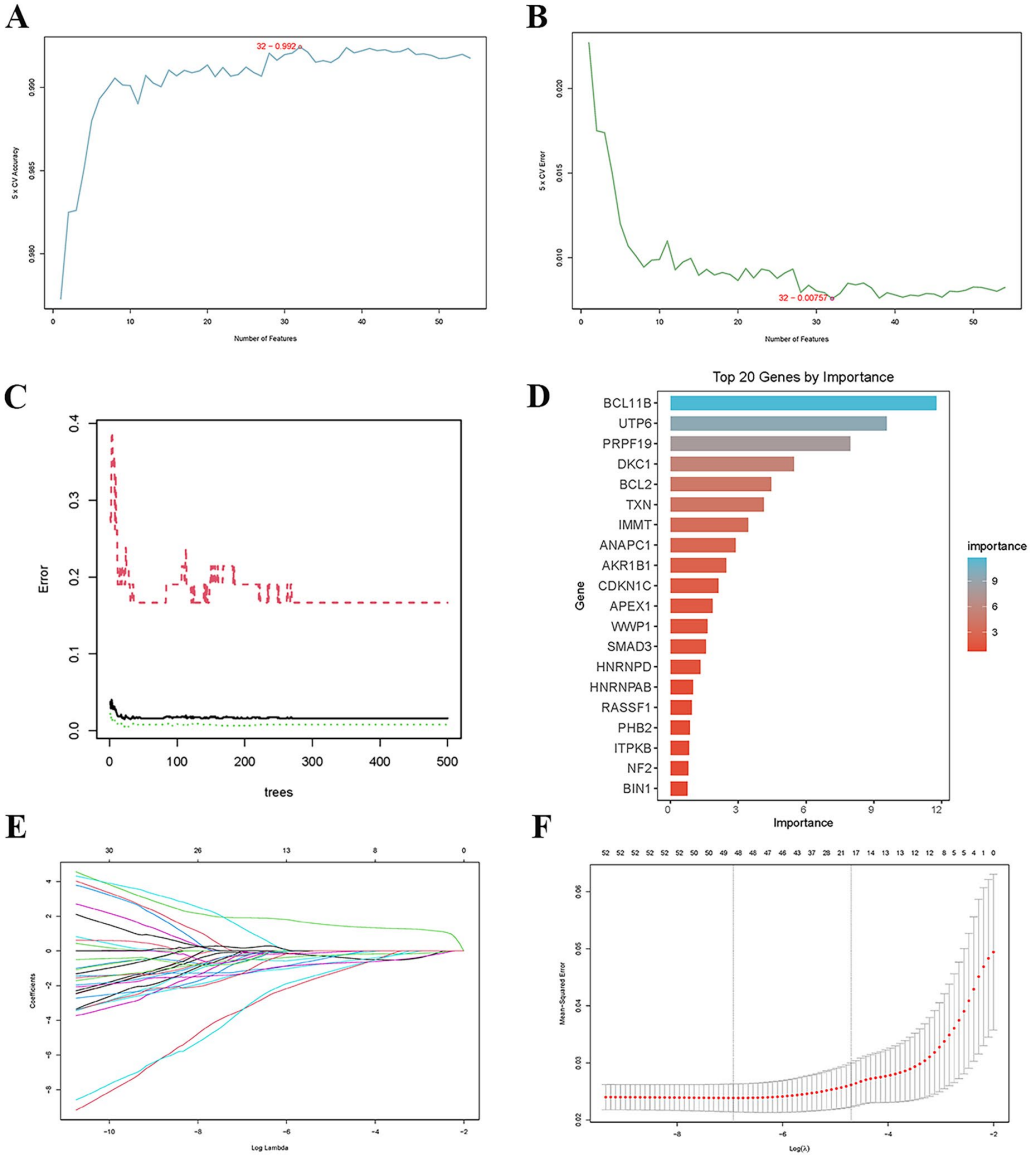
Screening of hub DE-SRGs using machine learning algorithms. (A, B) Feature selection using SVM-RFE. (A) Accuracy curve of SVM-RFE. (B) Error curve of SVM-RFE. (C, D) Feature selection using the RF algorithm. (C) Error rates of the random forest model. (D) The top 20 DE-SRGs for importance screened by RF algorithm. (E, F) Feature selection using LASSO regression. (E) LASSO coefficient profiles of the genes. (F) Ten-fold cross-validation for tuning parameter (*λ*) selection in the LASSO model. DE-SRGs: differentially expressed senescence-related genes; SVM-RFE: support vector machine-recursive feature elimination; RF: random forest; LASSO: least absolute shrinkage and selection operator.

**Figure 5.**
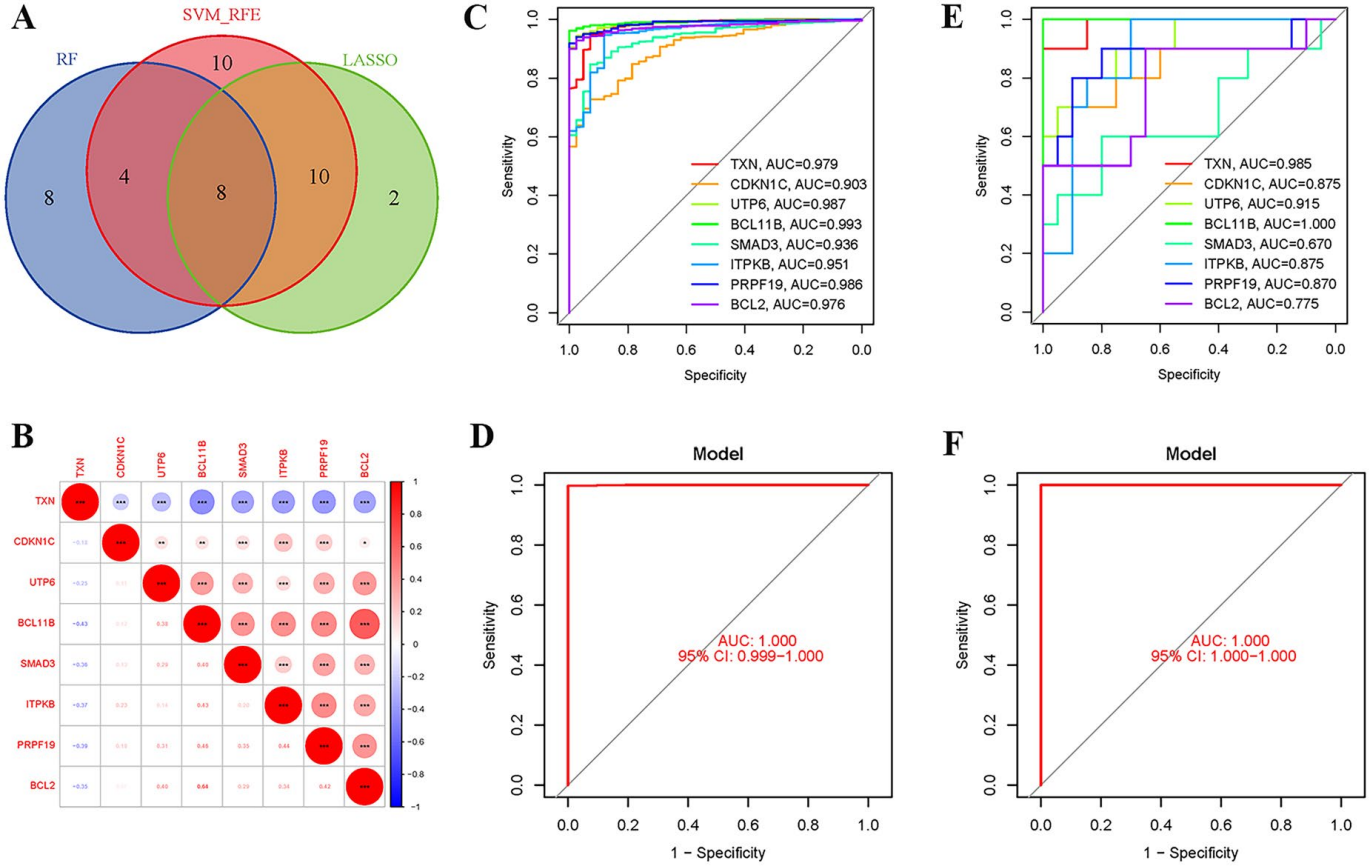
Identification and diagnostic validation of hub DE-SRGs. (A) Venn diagram illustrating the eight hub DE-SRGs identified by the intersection of three machine learning methods. (B) Correlation matrix depicting pairwise spearman correlation coefficients among the expression levels of the eight hub DE-SRGs. (C, D) Diagnostic performance evaluation in the training set. (C) ROC curves and corresponding AUC values for each individual hub gene. (D) ROC curve of the logistic regression model constructed using all eight hub DE-SRGs. (E, F) Diagnostic performance evaluation in the independent validation set. (E) The ROC curve for the diagnostic efficacy of individual genes. (F) The ROC curve for the diagnostic efficiency of the logistic regression model based on eight hub DE-SRGs. DE-SRGs: differentially expressed senescence-related genes; AUC: area under curve; ROC curves: receiver operating characteristic curves; CI: confidence interval.

In this study, three machine learning algorithms, namely RF, LASSO, and SVM-RFE, were adopted to further screen the hub DE-SRGs with diagnostic value from the above 54 DE-SRGs. The SVM-RFE findings revealed that the accuracy rate for 32 features was 0.992, with an error rate of 0.00757 (Figure 4(A,B)). The diagnostic errors for the RF method were shown, and the top 20 genes were ordered in descending order of importance (Figure 4(C,D)). Finally, LASSO regression identified 20 characteristic genes (Figure 4(E,F)). The Venn diagram demonstrated that three machine learning algorithms identified eight hub DE-SRGs, including TXN, CDKN1C, UTP6, BCL11B, SMAD3, ITPKB, PRPF19, and BCL2 (Figure 5(A)). Figure 5(B) displays the link between hub genes, with BCL11B having a substantial negative correlation with TXN and a significant positive correlation with BCL2. Figure 5(C) depicts the ROC curves of the eight hub DE-SRGs from the training set. The AUC for each gene surpassed 0.9, indicating considerable diagnostic impact. The logistic regression model based on these genes likewise had strong diagnostic results (AUC = 1.0, 95% CI: 0.999–1.00, Figure 5(D)). In the validation set, the eight hub DE-SRGs also showed high diagnostic performance. The AUC of seven genes was more than 0.7, while the AUC of the logistic regression model was 1 (Figure 5(E,F)).

### 3.4. The expression of hub DE-SRGs in the validation set

**Figure 6.**
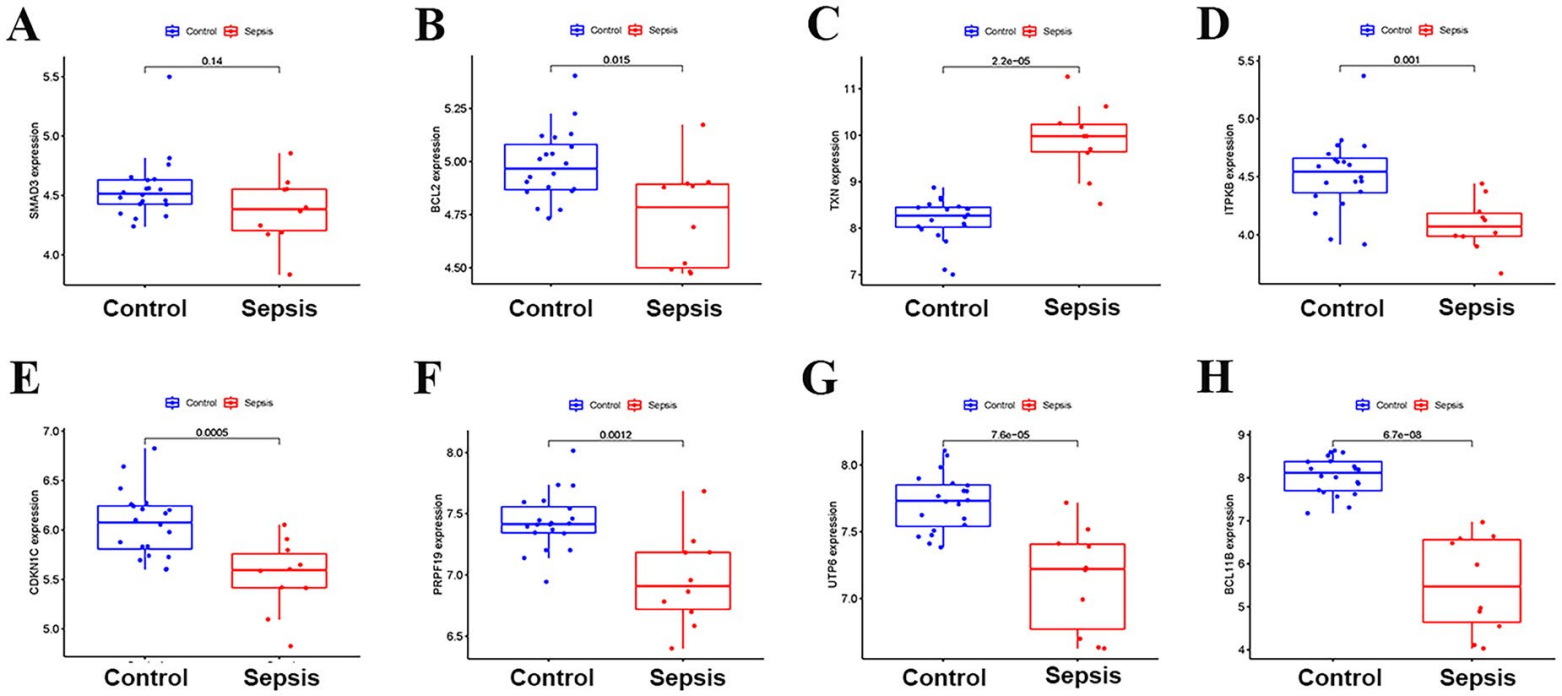
Differential expression analysis of hub DE-SRGs in the validation set. Box plots showing the mRNA expression levels of (A) SMAD3, (B) BCL2, (C) TXN, (D) ITPKB, (E) CDKN1C, (F) PRPF19, (G) UTP6, and (H) BCL11B in the validation dataset. Data are presented as mean ± SD. DE-SRGs: differentially expressed senescence-related genes; SD: standard deviation.

This study analyzed the expression trends of hub DE-SRGs in the validation set (Figure 6(A–H)). The results showed that compared with the control group, BCL2 (*p* = 0.015), ITPKB (*p* = 0.001), CDKN1C (*p* = 0.0005), PRPF19 (*p* = 0.0012), UTP6 (*p* = 7.6e − 05), and BCL11B (*p* = 6.7e − 08) decreased significantly in the sepsis group, while the expression of TXN (*p* = 2.2e − 5) increased significantly. There was no significant difference in SMAD3 between the two groups (*p* = 0.14).

### 3.5. Immunoinfiltration analysis

**Figure 7.**
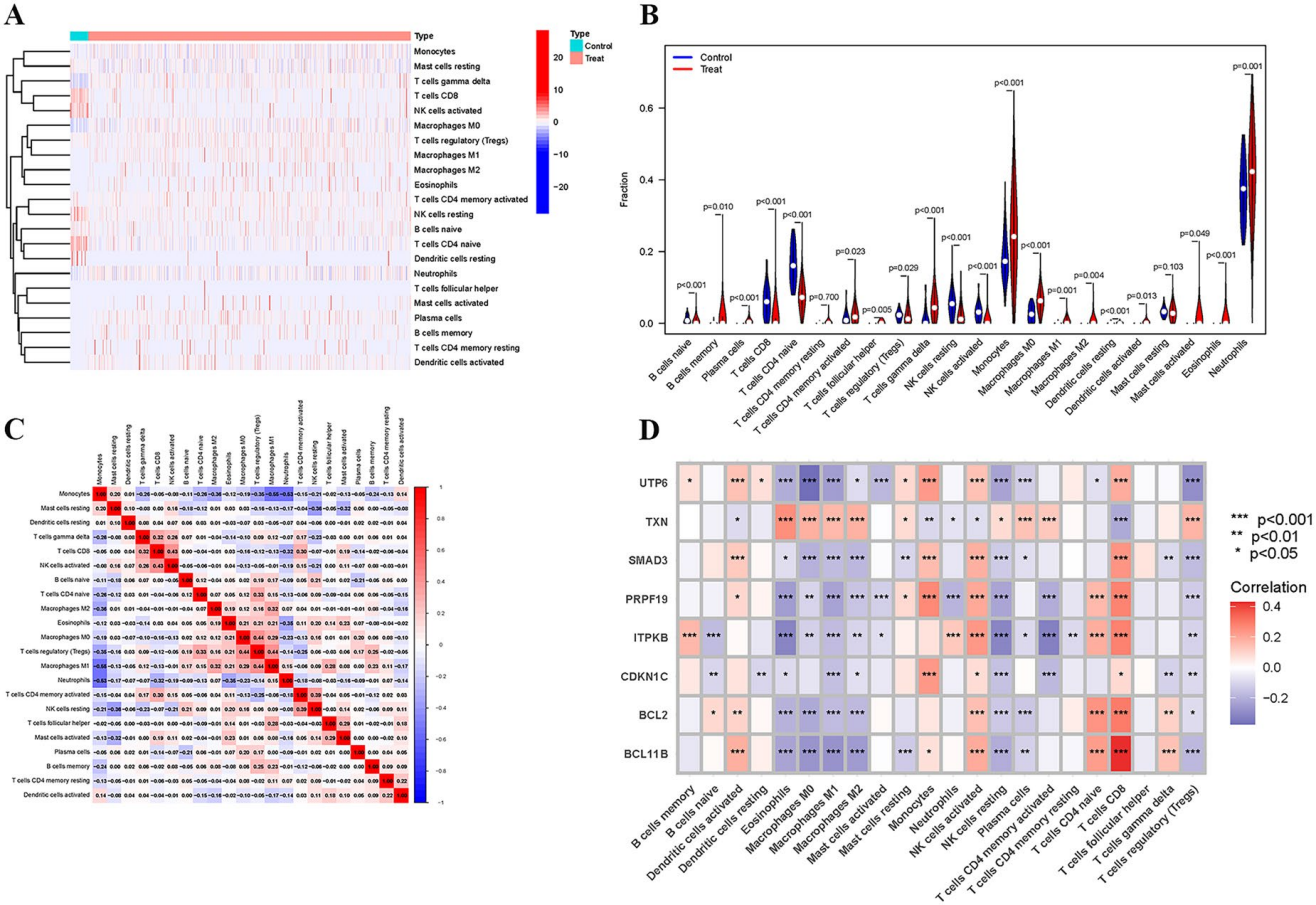
Analysis of immune cell infiltration and its association with hub DE-SRGs in sepsis. (A) Heatmap illustrating the relative abundance of 22 immune cell types across all samples in the training set. Each row represents an immune cell type, and each column corresponds to an individual sample from the training set. (B) Violin plot for differential analysis of immune cells in sepsis and healthy controls. (C) Correlation network among the 22 immune cell types. Red and blue lines represent positive and negative correlations, respectively. (D) Heatmap visualizing the correlation between the expression of the eight hub DE-SRGs and immune cells. DE-SRGs: differentially expressed senescence-related genes.

Sepsis could severely impair both the innate and adaptive immune systems of patients. In the initial stage, innate immune cells recognized pathogen-associated molecular patterns (PAMPs) and damage-associated molecular patterns (DAMPs) to activate immune responses, cleared pathogens, and presented antigens to adaptive immune cells. However, the inflammatory storm was followed by immunosuppression, thereby increasing the risk of secondary infection. The CIBERSORT approach was employed in this study to analyze the sepsis immunological microenvironment. The analysis was performed using the CIBERSORT algorithm with the normalized gene expression matrix of the training cohort (GSE65682) as the input mixture file (Table S7). The LM22 leukocyte gene signature matrix (Table S8), containing 547 marker genes defining 22 human immune cell phenotypes, was used as the reference. The algorithm was run with 1,000 permutations for significance estimation, and quantile normalization was disabled as recommended for normalized microarray data. Figure 7(A) shows the abundance of 22 immune cell types in each sample of the training set.

Figure 7(B) compares the expression of these immune cells in the sepsis and control groups. Monocytes and neutrophils made up a large fraction of both groups, with the sepsis group having much higher levels. Meanwhile, CD8+ T cells and naïve CD4+ T cells were significantly decreased in the sepsis group. Correlation study revealed a strong positive relationship between activated NK cells and CD8 T cells, as well as M0 macrophages and Tregs. Monocytes exhibited a strong negative connection with M1 macrophages and neutrophils (Figure 7(C)). This study looked more into the relationship between hub DE-SRGs and immune cells. Among them, eosinophils, macrophages M1, macrophages M2, NK cells activated, NK cells resting, T cells CD8, and Tregs were substantially linked with all hub DE-SRGs, indicating that these immune cells might play an essential role in sepsis (Figure 7(D)).

### 3.6. Targeted drugs and molecular docking for hub genes

**Figure 8.**
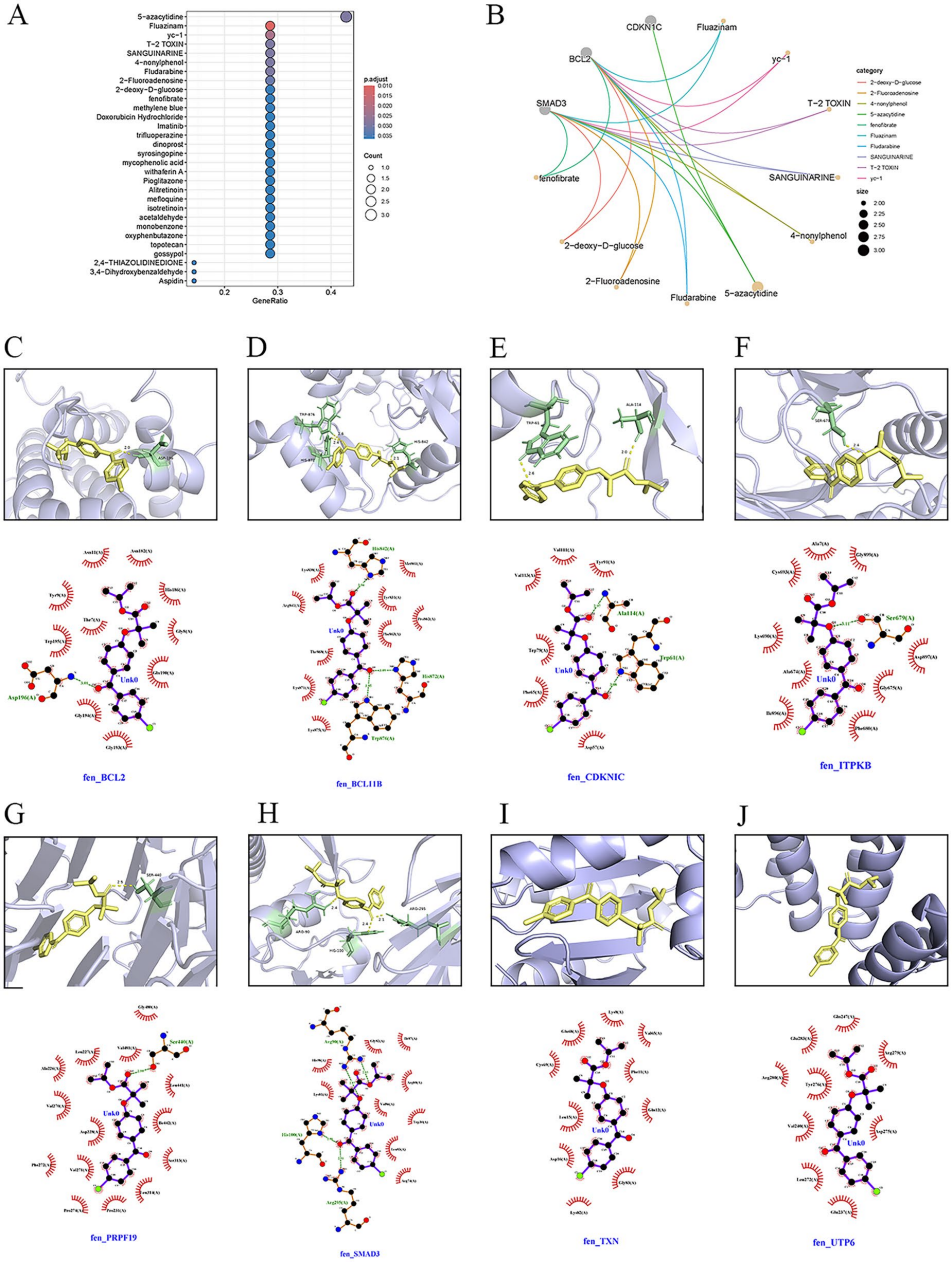
Drug enrichment analysis and molecular docking. (A) Bar plot displaying the top 30 candidate drugs or compounds most significantly enriched for the hub genes. (B) Drug–gene interaction network between the hub DE-SRGs and the top 10 enriched drugs. (C–J) Molecular docking models of fenofibrate with the protein products of the hub DE-SRGs. For each target (C) SMAD3, (D) BCL2, (E) TXN, (F) ITPKB, (G) CDKN1C, (H) PRPF19, (I) UTP6, and (J) BCL11B, the up panel shows the 3D binding pose, and the down panel illustrates the 2D ligand–protein interaction diagram. DE-SRGs: differentially expressed senescence-related genes.

Targeted drugs for hub DE-SRGs were retrieved from the DSigDB database (https://dsigdb.tanlab.org/DSigDBv1.0/). Figure 8(A) illustrates the top 30 drugs exhibiting the most significant hub gene enrichment. These drugs primarily interacted with CDKN1C, BCL2, and SMAD3 (Figure 8(B)). Fenofibrate, a commonly used lipid-lowering agent, was a classic PPARα agonist. PPARα was extensively expressed in the liver, kidneys, heart, and skeletal muscle. It enhanced fatty acid oxidation, controlled energy metabolism, and had anti-inflammatory properties. This study selected fenofibrate for further investigation. AutoDock Vina was used to mimic the drug’s binding to the target protein. PyMOL (v3.1.1) and Ligplot (v2.2.5) were used to depict intermolecular interactions such as hydrogen bonding, hydrophobic contact, and others. Key residues involved in binding were identified to elucidate the molecular basis of the interaction. Figure 8(C–J) shows the interactions between fenofibrate and eight target proteins, respectively. The binding energies of fenofibrate with these target proteins were −7.3 kcal/mol (SMAD3), −7.3 kcal/mol (BCL2), −5.3 kcal/mol (TXN), −7.3 kcal/mol (ITPKB), −7.2 kcal/mol (CDKN1C), −7.6 kcal/mol (PRPF19), −6.7 kcal/mol (UTP6), and −7.1 kcal/mol (BCL11B), all of which were less than −5.0 kcal/mol, indicating that fenofibrate could form stable bonds with these proteins.

### 3.7. Fenofibrate reduced cellular senescence by targeting critical senescence genes in sepsis

**Figure 9.**
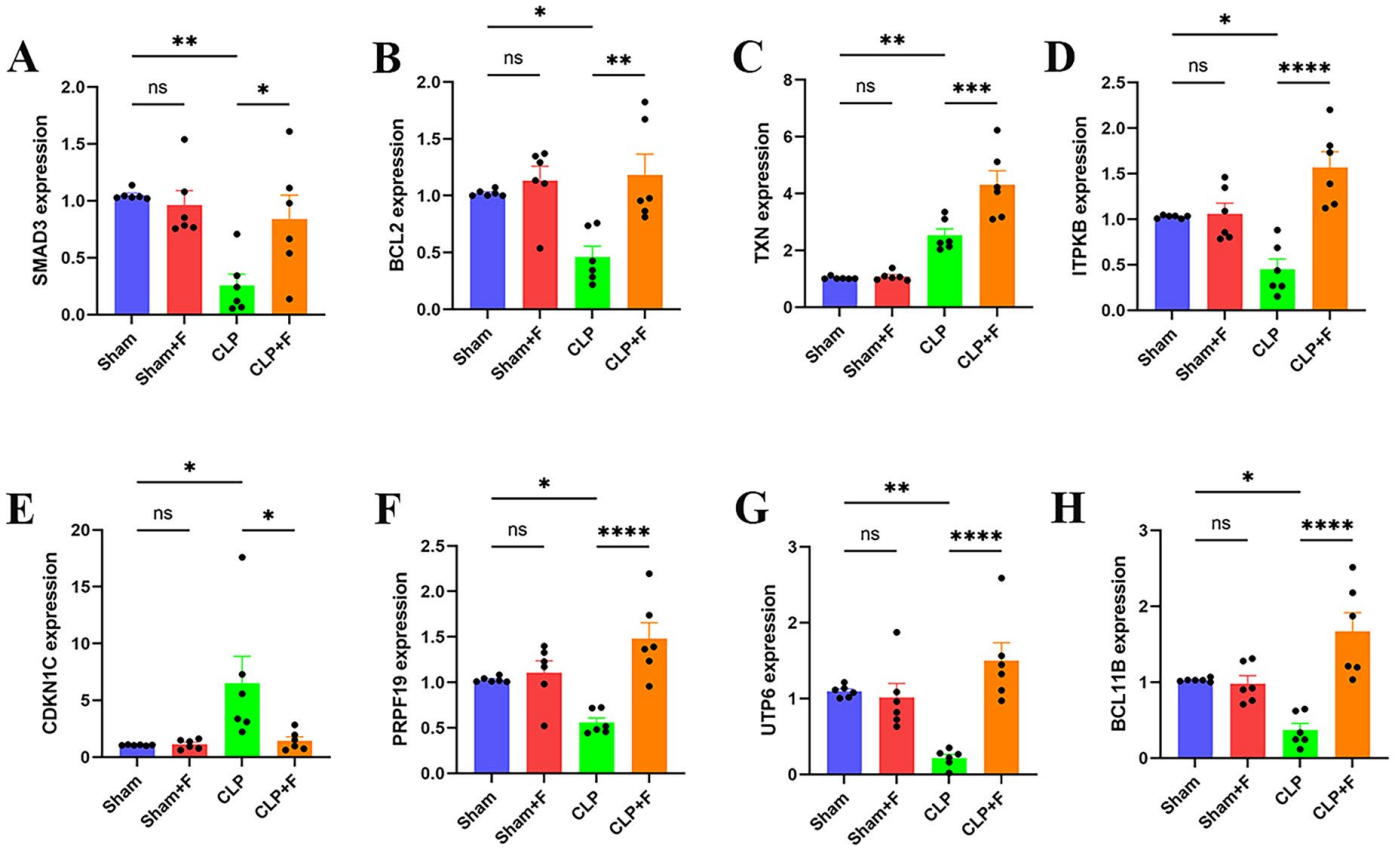
Fenofibrate modulates the expression of hub DE-SRGs in septic kidney injury. RT-qPCR analysis of mRNA expression levels for (A) SMAD3, (B) BCL2, (C) TXN, (D) ITPKB, (E) CDKN1C, (F) PRPF19, (G) UTP6, and (H) BCL11B in renal tissues from the indicated experimental groups (*n* = 6). Data are presented as mean ± SD. ns: *p* > 0.05; *p < 0.05; **p < 0.01; ***p < 0.001; ****p < 0.0001. DE-SRGs: differentially expressed senescence-related genes; SD: standard deviation; ns: not significant.

**Figure 10.**
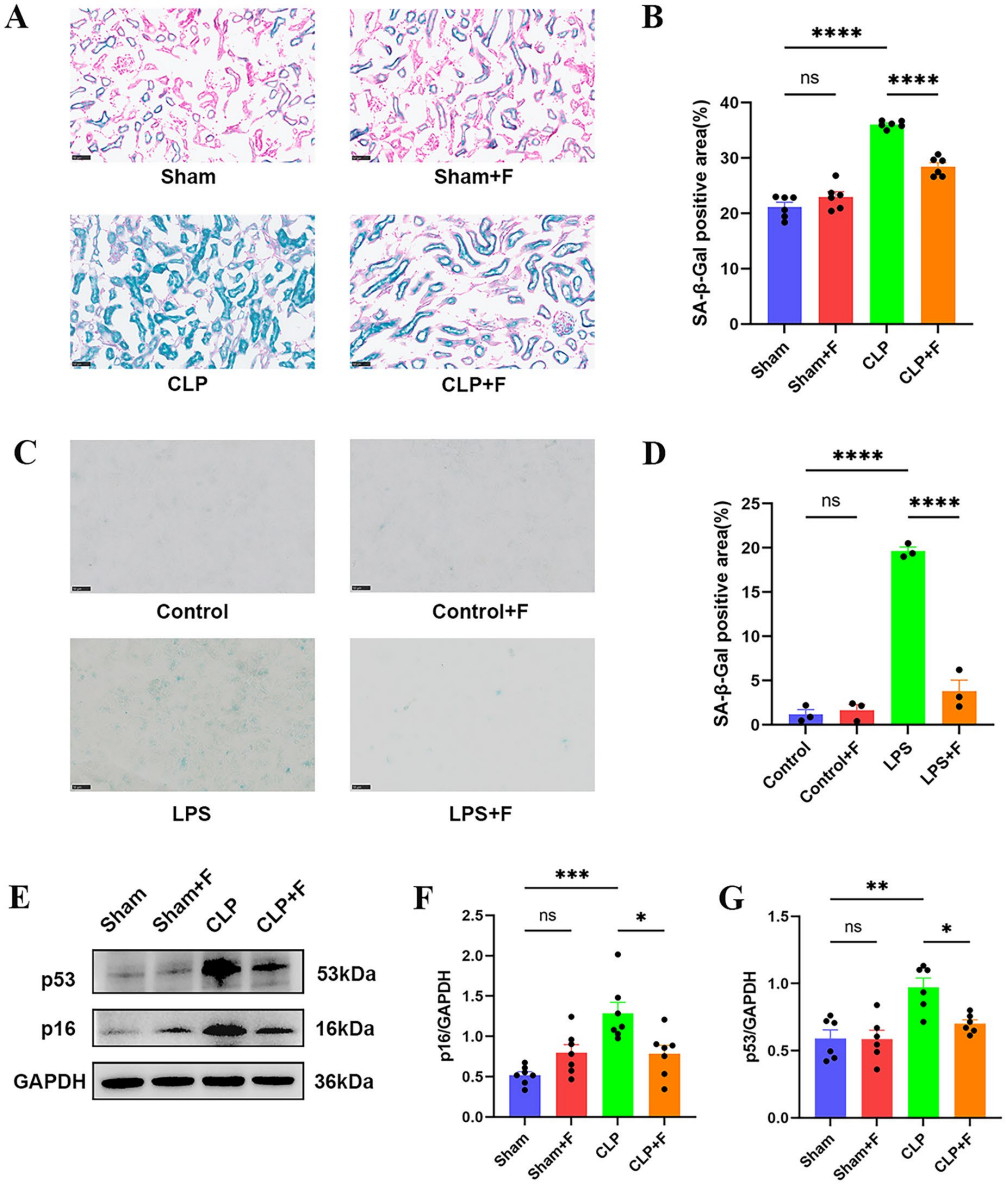
Fenofibrate mitigates cellular senescence in sepsis-associated acute kidney injury. (A) Representative images of SA-β-Gal staining in kidney tissue sections from different experimental groups (scale bar = 50 µm). (B) Quantification of SA-β-Gal-positive area in renal tissues (*n* = 6). (C) Representative images of SA-β-Gal staining in HK-2 cells treated as indicated (scale bar = 50 µm). (D) Quantification of SA-β-Gal-positive area in HK-2 cells (*n* = 3). (E) Representative western blot images showing protein levels of p53, p16, and GAPDH in renal tissues. (F, G) Quantitative analysis of (F) p53 and (G) p16 protein expression normalized to GAPDH (*n* = 6). Data are presented as mean ± SD. ns: *p* > 0.05; *p < 0.05; **p < 0.01; ***p < 0.001; ****p < 0.0001. SA-β-Gal: senescence-associated β-galactosidase; ns: not significant; SD: standard deviation.

This study administered fenofibrate to septic mice and investigated its influence on key senescence genes in sepsis. There were no significant differences in the eight hub DE-SRGs between Sham and Sham + F group. In the CLP group, mRNA expression levels of SMAD3, BCL2, ITPKB, PRPF19, UTP6, and BCL11B were considerably lower than in the Sham group, but rose dramatically following fenofibrate therapy. TXN and CDKN1C mRNA expression levels were higher in the CLP group. TXN expression climbed during fenofibrate therapy, but CDKN1C expression decreased (Figure 9). SA-β-Gal was a reliable biomarker of cellular senescence. We detected SA-β-Gal activity in both *in vivo* and *in vitro* models of sepsis-induced AKI in this study. SA-β-Gal activity was greatly enhanced in septic mice’s kidneys, but reduced following fenofibrate administration (Figure 10(A,B)). Consistent results were also obtained *in vitro* models of sepsis-induced AKI (Figure 10(C,D)). p53 and p16 were typical cellular senescence proteins that were considerably raised in septic mouse kidney tissue but dramatically lowered following fenofibrate therapy (Figure 10(E–G)). These findings suggested that fenofibrate could target key senescence genes in sepsis and reduce cellular senescence in sepsis-induced AKI.

### 3.8. Fenofibrate could improve sepsis induced AKI

**Figure 11.**
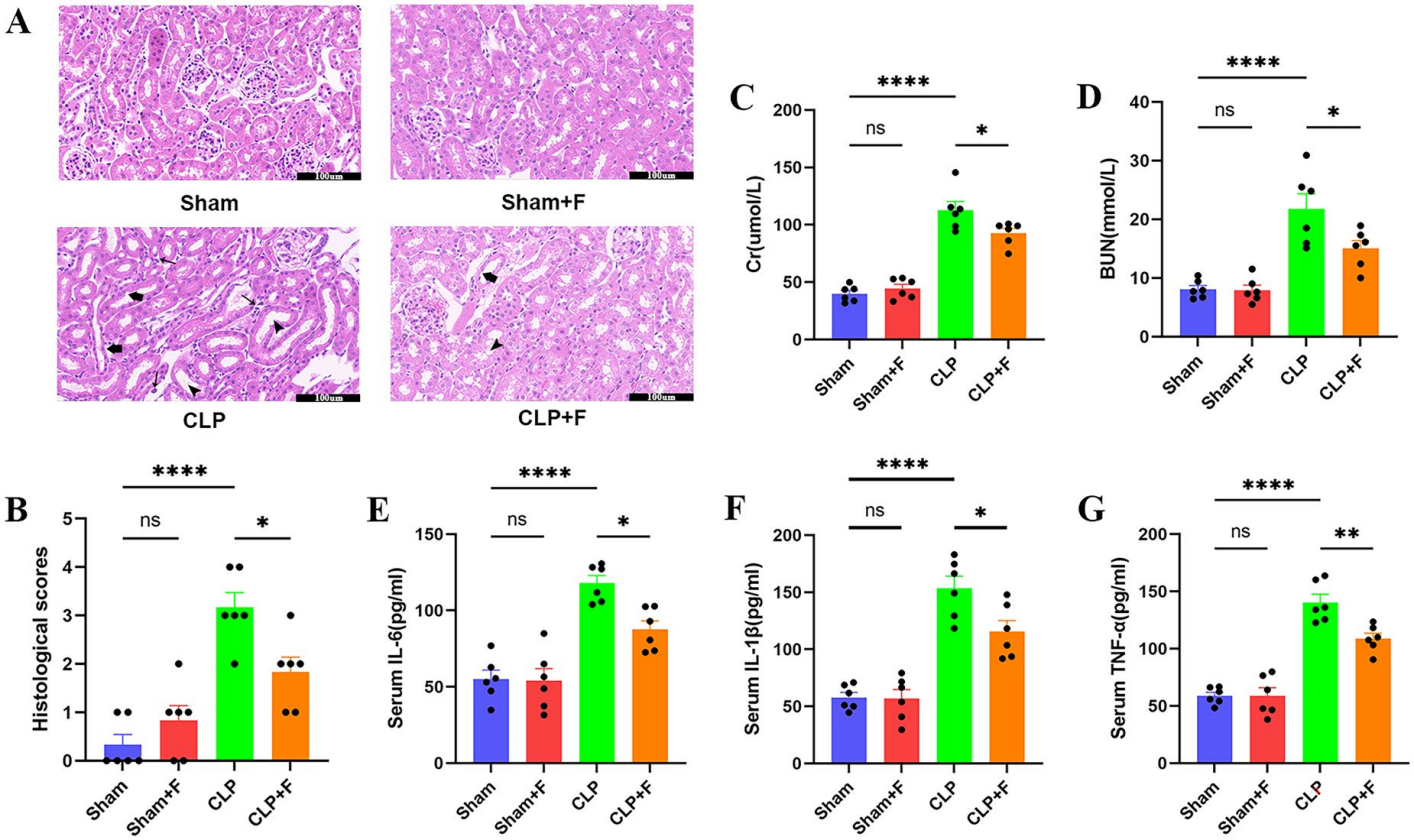
Fenofibrate ameliorates renal injury and systemic inflammation in septic mice. (A) Representative HE-stained sections of kidney tissues (scale bar = 100 μm). Pathological features are annotated: thin arrows indicate inflammatory cell infiltration; thick arrows indicate tubular epithelial damage and detachment; arrowheads indicate tubular dilation and swelling (*n* = 6). (B) Semiquantitative histopathological injury scores of renal tissues (*n* = 6). (C, D) Serum levels of (C) creatinine and (D) BUN (*n* = 6). (E–G) Serum concentrations of pro-inflammatory cytokines (E) IL-6, (F) IL-1β, and (G) TNF-α (*n* = 6). Data are presented as mean ± SD. ns: *p* > 0.05; *p < 0.05; **p < 0.01; ****p < 0.0001. BUN: blood urea nitrogen; IL-6: interleukin-6; IL-1β: interleukin-1β; TNF-α: tumor necrosis factor-α; SD: standard deviation.

This study further evaluated the therapeutic effects of fenofibrate on sepsis-induced AKI. Fenofibrate was found to considerably ameliorate pathological damage of renal tissue in septic mice using HE staining (Figure 11(A,B)). The levels of creatinine and urea nitrogen, which were commonly used as indicators of kidney injury, decreased significantly in septic mice treated with fenofibrate (Figure 11(C,D)). This study also detected the expressions of IL-6, IL-1β, and TNF-α in serum to reflect the inflammatory level. The findings revealed that inflammatory variables were considerably elevated in septic mice, but dramatically reduced following treatment with fenofibrate (Figure 11(E–G)). These findings implied that fenofibrate had anti-inflammatory properties and could protect against sepsis-induced AKI.

## 4. Discussion

The pathophysiological mechanisms of sepsis were intricate and, at present, there was no specific treatment available. This study identified eight hub DE-SRGs (SMAD3, BCL2, TXN, ITPKB, CDKN1C, PRPF19, UTP6, and BCL11B), six of which (BCL2, TXN, ITPKB, PRPF19, UTP6, and BCL11B) yielded consistent results in the validation set, human samples, and mouse samples. All of these genes had good diagnostic efficacy for sepsis. Further analysis of immune infiltration revealed that these hub DE-SRGs were closely associated with macrophages, NK cells, CD8 T cells, and Tregs. Finally, the protective effect of fenofibrate against sepsis-induced AKI was verified *in vivo* and *in vitro* models after targeted drugs were screened in the DSigDB database.

BCL2 was an important anti-apoptotic protein belonging to the Bcl-2 protein family. The way it worked might be connected to preventing the release of cytochrome c from mitochondria and/or binding to apoptosis-activating factor (APAF-1), which in turn inhibited caspase activity [19]. Numerous studies had demonstrated that sepsis led to increased cell apoptosis [20–22]. The high mortality of sepsis was closely tied to post-sepsis immune paralysis. It was considered that uncontrolled apoptosis-induced alterations to immune cells were the primary cause of severe immunosuppression. Lymphocyte apoptosis, in particular, was associated with an enhanced risk of secondary infection and worse prognosis. This study revealed that BCL2 expression was notably reduced in patients with sepsis, indicating a substantial inhibition of anti-apoptotic activity.

Thioredoxin (TXN or Trx) together with thioredoxin reductase (TR) and reduced nicotinamide adenine dinucleotide phosphate (NADPH) constituted the thioredoxin system. Previous studies had shown that the thioredoxin system played an important role in diseases such as cancer, neurodegenerative diseases, osteoarthritis, and sepsis, with mechanisms involving ferroptosis [23,24], cellular senescence [25], endoplasmic reticulum stress [26], and m6A methylation [27]. TXN was an essential antioxidant molecule that facilitated redox reactions and defended against cell death caused by various factors. TXN had three subtypes, namely TXN1, TXN2, and TXN3, which were mainly found in the cytoplasm, mitochondria, and testicles, respectively. Bai et al. discovered that TrxR1 regulated the redox state of TXN-1, which governed the internalization of bacterial outer membrane vesicles (OMVs) and caspase-11 activation. These findings implied that cellular redox stress might activate caspase-11 as an inflammatory signal in sepsis. Furthermore, TXN was also valuable for the diagnosis of sepsis. The results of bioinformatics analysis conducted by Zhou et al. revealed that the AUC of TXN in the sepsis training set was 0.983, and in the validation set, it was 0.736 [26]. Another study showed that TXN achieved an AUC value of 0.982 in the sepsis training set and 0.853 in the validation set [27]. This study found that TXN expression was much higher in the sepsis group and had strong diagnostic performance for sepsis, which is consistent with earlier researches.

ITPKB controlled inositol phosphate metabolism by phosphorylating the second messenger, inositol 1,4,5-trisphosphate, which produced Ins (1,3,4,5) P4. ITPKB activity was modulated by calcium/calmodulin and protein phosphorylation mechanisms. Li et al. observed that in ovarian cancer, CAMK2G, a CAMKII isoenzyme, could detect basal and cisplatin-induced ROS, causing ITPKB to be phosphorylated at the serine S174 site and directly regulating ITPKB activity to modulate cisplatin-induced ROS stress, which was closely related to platinum resistance [28]. ITPKB had also been linked to a bad prognosis in glioblastoma patients. Mechanistically, reduced phosphorylation of E3 ligase Trim25 in recurrent glioblastoma multiforme samples led to weakened ITPKB ubiquitination and increased ITPKB stability, thereby inhibiting ROS production. In addition, the ITPKB inhibitor or ITPKB depletion significantly overcame TMZ chemoresistance in a glioblastoma xenograft mouse model [29]. There was little investigation into ITPKB in sepsis. In this study, ITPKB expression levels were considerably lowered in sepsis samples, which might be attributed to redox imbalance in sepsis.

PRPF19 was a key component of the Prp19 complex, which was located within the spliceosome and played an important role in its assembly, remodeling, and activity. PRPF19 was involved in regulating oxidative stress [30,31] and alleviating cell apoptosis and senescence [32,33]. Yano et al. discovered that PRPF19 increased MDM4-mediated p53 inactivation in normal human diploid fibroblasts, resulting in an anti-cell senescence effect. Knocking down PRPF19 caused MDM4 splicing isoforms to transform into the unstable MDM4-S lacking exons, which induced cell cycle arrest [34]. However, high expression of PRPF19 in hepatocellular carcinoma (HCC) was associated with poor prognosis [35]. Deubiquitinated PRPF19 enhanced migration and invasion in hepatocellular cancer [36]. PRPF19 had also been demonstrated to enhance tongue cancer cell proliferation, migration, and development, as well as resistance to radiation and cisplatin treatment [37]. PRPF19 also functioned as an oncogene in pancreatic cancer and could be used as an independent biomarker for diagnosis and prognosis of the disease [38]. This study found that PRPF19 levels were significantly lower in sepsis samples, which suggested that cellular senescence was widespread.

UTP6, also known as hepatocellular carcinoma antigen 66 (HCA66), was the first precursor of the small ribosomal subunit in eukaryotes. Fant et al. claimed that HCA66 was a novel regulator of gamma-tubulin function that stabilized components of the gamma-tubulin small complex, which was required for the assembly of the larger gamma-tubulin ring complex [39]. According to Piddubnyak et al. neurofibromatosis type 1 (NF1) cells with haploinsufficiency-derived UTP6 underexpression were less prone to apoptosis [40]. It was additionally thought to be a component of the human histone deacetylase family. These studies demonstrate the wide range of functions of UTP6.

BCL11B was a C2H2-type zinc finger protein that regulated the differentiation and survival of T cells in the mammalian thymus. It modulated CCR7 and CCR9 expression to govern how hematopoietic stem cells respond to chemotactic signals, directing precursor cells from the bone marrow to the thymus [41,42]. BCL11B was also involved in a variety of disorders. Huang et al. discovered that has-miR-150 targeting BCL11B might be associated with the pathophysiology of newborn sepsis using expression profile data. Another study had revealed that the lack of BCL11B accelerated mammary aging and increased DMBA-induced tumor development [43]. This study discovered that BCL11B was considerably lower in the sepsis group than in the control group, which might accelerate cellular senescence in sepsis.

SMAD3 was a core transcriptional regulatory factor in the transforming growth factor-β (TGF-β) signaling pathway, belonging to the receptor-modulating SMAD (R-SMAD) of the SMAD protein family. It was involved in several processes, including cell proliferation, differentiation, apoptosis, immunological modulation, fibrosis, and carcinogenesis. According to Liu et al. large levels of SMAD3 were crucial in causing cellular senescence outside of the p53/p16/p15 senescence pathways when the oncogene Ras was overactivated in lung epithelial cells. SMAD3 formed a transcriptional complex with the putative tumor suppressor ATOH8, directly blocking the expression of a number of genes that derived cell cycle progression, resulting in senescence in lung epithelial cells [44]. Gauthier et al. discovered that activating SMAD3 in macrophages in an activin A-dependent manner reduced inflammation in mice with psoriasis or sepsis [45]. In this work, it was possible to conclude that lower SMAD3 expression in sepsis samples was associated with defective anti-inflammatory responses and accelerated cellular senescence.

CDKN1C is a potent inhibitor of various CDK complexes, including cyclin A-CDK2, cyclin D2-CDK4, and cyclin E-CDK2, and is thought to be a negative regulator of cell growth, perhaps contributing to a lifelong non-proliferative state. CDKN1C was intimately associated with senescence. A research on HCC has shown that Hes1 could target and inhibit CDKN1C. Downregulating Hes1 caused HCC cells to flatten and elongate, accumulate age-related β-galactosidase, and enhance P16 expression [46]. CDKN1C had substantial diagnostic relevance in sepsis patients. Yang et al. classified sepsis patients into high-aging and low-aging groups based on gene set variation analysis (GSVA) scores derived from the GOBP-AGING gene set and found that ARG1/CDKN1C appeared to be an effective biomarker for the early diagnosis of sepsis patients, with an AUC of 0.996 [47]. In this study, CDKN1C levels were decreased in sepsis samples in both the training and validation sets, whereas the trend in the experiment was opposite. This may be related to species heterogeneity and disease progression.

Our immune infiltration analysis revealed distinct correlation patterns between hub DE-SRGs and immune cell subsets in sepsis patients. Specifically, TXN showed positive correlations with eosinophils, macrophages, resting NK cells, and Tregs, while exhibiting negative correlations with activated NK cells and CD8 T cells. In contrast, the remaining seven hub genes (BCL2, BCL11B, SMAD3, ITPKB, CDKN1C, PRPF19, and UTP6) displayed opposing trends: positive associations with activated NK cells and CD8 T cells, but negative correlations with the aforementioned subsets (eosinophils, macrophages, resting NK cells, and Tregs). These findings suggest that TXN may promote an immunosuppressive phenotype in sepsis by maintaining redox homeostasis and enhancing antioxidant capacity, thereby supporting the survival and function of immunosuppressive cells such as Tregs and M2 macrophages to attenuate excessive inflammatory responses. This notion is supported by previous studies demonstrating analogous mechanisms in other contexts. For example, in metastatic melanoma models, TXN induces Treg recruitment, thereby establishing an immunotolerant tumor microenvironment and facilitating immune evasion [48]. Furthermore, human natural Tregs express and secrete elevated levels of TXN-1, conferring enhanced tolerance to oxidative stress and sustaining their suppressive activity [49]. TXN-1 has also been shown to promote macrophage polarization toward the anti-inflammatory M2 phenotype, reinforcing immunosuppressive responses [50]. Such mechanisms may similarly contribute to the biphasic immune dynamics characteristic of sepsis, characterized by an initial hyperinflammatory phase followed by a compensatory immunosuppressive state. However, these observations are derived from correlative analyses and do not establish causality. Previous studies have reported multiple genes closely associated with the immune microenvironment and prognosis in sepsis, offering valuable directions for further elucidating the mechanisms underlying TXN-driven immunosuppression [51,52].

This study searched the DSigDB database for drugs targeting hub genes. Fenofibrate was a widely utilized lipid-modulating agent that activated PPARα, a nuclear receptor, to enhance the production of lipoprotein lipase, apolipoprotein AI, and apolipoprotein AII genes, while suppressing the expression of apolipoprotein CIII gene and fatty acid β-oxidation. This facilitated the elimination of triglyceride-rich lipoproteins, reduced plasma cholesterol, and elevated high-density lipoprotein levels. Furthermore, prior research had demonstrated that fenofibrate could modulate inflammatory responses alongside its regulation of blood lipids. Tancevski et al. observed that fenofibrate had the capacity to reduce systemic inflammatory responses in mice with sepsis. LPS mechanistically increased the activation of mitogen-activated protein kinase (ERK), resulting in the downregulation of G protein-coupled receptor kinase-2 (GRK2) and chemokine receptor CXCR2, thereby limiting neutrophil chemotaxis. Fenofibrate counteracted this process [53]. In addition, fenofibrate had been observed to enhance renal function, reduced proteinuria, and reversed histological changes, including glomerulosclerosis and tubulointerstitial fibrosis, in aged mice. This advantageous effect pertained to the AMPK and SIRT1 signaling pathways. Consequently, the targeting of PPARα and the AMPK–SIRT1 signaling pathway might represent a viable approach to mitigating age-related renal damage [54]. Fenofibrate had also been shown to protect against AKI through processes involving eNOS expression and energy metabolism [55–57]. This study found that fenofibrate had anti-inflammatory and anti-senescent effects in the mouse model of AKI, which could be connected to fenofibrate’s modulation of senescence-associated genes. These findings provided a theoretical basis for using fenofibrate to treat sepsis.

There are several limitations to this study. First, although this study identified multiple SRGs that were differentially expressed in sepsis and validated in mouse samples, further exploration was required to elucidate the underlying mechanisms of these genes in the progression of sepsis. Second, this study was mostly based on the analysis of expression profile data, and more clinical data needed to be collected to corroborate the link between hub DE-SRGs and patient clinical features. Third, this study only confirmed fenofibrate’s protective effect on AKI in mice. Additional research would be necessary to determine its effects on other organs.

## 5. Conclusions

The present study identified eight hub DE-SRGs associated with sepsis and validated their diagnostic efficacy for the condition. Immunological microenvironment analysis demonstrated a close relationship between hub DE-SRGs and several immune cell types, including macrophages, NK cells, CD8 T cells, and Tregs. Finally, this study identified fenofibrate’s anti-inflammatory and anti-senescence properties in sepsis-associated AKI, shedding fresh light on sepsis treatment.

## Supplementary Material

Supplemental Material

Supplemental Material

Supplemental Material

Supplemental Material

Supplemental Material

Supplemental Material

Supplemental Material

Supplemental Material

## Data Availability

The datasets supporting the findings of this study have been deposited in the Zenodo repository and are accessible via the following DOIs: 10.5281/zenodo.18361082; 10.5281/zenodo.18948938.
